# Electricity in Electrotherapeutics

**Published:** 1896-07

**Authors:** 


ELECTRICITY IN ELECTROTHERAPEUTICS. By EDWIN J. HOUSTON, PH. D., and A. E. KENNELLY, Sc. D. Price, $1.00. New York: The W. J. Johnston Company, 253 Broadway. 1896.

This little volume of 402 pages gives in a concise form the fundamental principles of electricity. It describes at length the various cells in use with special reference to their advantages and disadvantages for medical purposes. This chapter and the following one on the laws governing the strength of current are especially commendable. The various induction coils, kinds of static machines, ammeters, commutators, cautery knives and incandescent lamps now in use, are fully discussed. The special electrodes and apparatuses for topical application are not described; in fact, the book is purely technical and does not enter into exact methods of therapeutical application. It is written clearly and concisely, and fulfils the purpose for which it was written. It is printed on good paper and contains 128 illustrations that are well executed. H. K.

